# The impact of caregiving on the roles and valued activities of stroke carers: A systematic review of qualitative studies

**DOI:** 10.1371/journal.pone.0304501

**Published:** 2024-05-31

**Authors:** Melissa Jammal, Gregory S. Kolt, Karen P. Y. Liu, Nariman Dennaoui, Emma S. George

**Affiliations:** 1 School of Health Sciences, Western Sydney University, Sydney, NSW, Australia; 2 Department of Rehabilitation Sciences, The Hong Kong Polytechnic University, Hong Kong SAR, Hong Kong; 3 Translational Health Research Institute, Western Sydney University, Sydney, NSW, Australia; Universidad Internacional de La Rioja, SPAIN

## Abstract

**Objective:**

To understand the experiences of informal carers and the impact of role and activity changes on their health and wellbeing.

**Methods:**

A systematic search of CINHAL, MEDLINE, Embase, APA PsycInfo, and Web of Science was conducted. Studies were eligible if they included informal stroke carers (≥18 years), used a qualitative methodology, explored the roles and valued activities of stroke carers, and were published in English. The 10-item Critical Appraisal Skills Programme checklist for qualitative studies was used to assess methodological quality. The results of the included studies were thematically synthesised.

**Results:**

A total of 36 qualitative studies were included and four overarching themes were identified: (1) Life adjustment; (2) Changing role and identity; (3) Changing activities: From meaningful to purposeful; and (4) Understanding and supporting carers.

**Conclusion:**

The sudden nature of stroke requires major readjustment in the carers life that has implications on their relationships, roles, and activities, subsequently impacting on their health and wellbeing. Health professionals and researchers should collaborate with stroke carers to identify their valued activities and implement realistic strategies to maintain these activities. Future interventions designed for carers should implement education about the importance of participating in valued activities and strategies to maintain these activities.

## Introduction

Globally, stroke is one of the leading causes of neurological disability in adults [1]. Following a stroke, many survivors will require assistance from carers throughout their recovery with rehabilitation and execution of daily activities [2]. An informal carer is an individual who provides unpaid support and care within the context of an existing relationship such as a family member, partner, neighbour, or friend [3]. Informal carers play a crucial role in providing ongoing practical and emotional support to meet the needs of stroke survivors [4, 5]. The demands of caregiving, however, can often result in the carer’s loss of independence, uncertainty of their role, feeling unsupported, and having difficulty accessing appropriate services [6–8].

Carers’ participation in valued activities such as leisure, social participation, and employment has been linked with improved wellbeing [9–11], while also providing structure and routine, contributing to the carer’s sense of identity and sense of connectedness with others [10]. The demands of caregiving, however, can limit the time carers have to participate in their valued activities, with many frequently reporting a loss of identity due to fewer opportunities to engage in their previous valued roles or activities [10, 12].

To date, there is little research examining the valued activities of stroke carers and the impact of these activities on their health and wellbeing [13–15]. The majority of studies in this area have focused primarily on the stroke survivors’ participation and performance of activities with a limited focus on the carer [16–19]. In addition, previous reviews demonstrate that carer interventions rarely address the loss of valued activities [20, 21], rather addressing outcomes such as carer burden, wellbeing, and quality of life [20].

A mixed methods systematic review by Pellerin et al. [15] described the challenges of relatives of stroke survivors in completing their daily activities. This review found that relatives of stroke survivors often have increased responsibilities and experience a reduction in leisure and interpersonal relationships. Although this review provides insight into the social participation of relatives of stroke survivors, the authors acknowledge that not all relatives are informal carers. In addition, the impact of caregiving on informal carers’ roles and valued activities such as employment and leisure activities remains unclear.

More recently, Jellema et al. [22] conducted a mixed methods scoping review to identify existing research on stroke carers’ valued activities and how caregiving impacts these activities. This review found that informal carers engage less in their valued activities such as social and leisure activities, resulting in lower levels of wellbeing, greater social isolation, and depression. The study also identified that there were no controlled trials that examined how caregiving, activity loss, and wellbeing are interrelated. Although the findings of this scoping review highlight carers’ reduced participation in valued activities, it remains unclear what strategies may assist carers to maintain their valued activities. The authors recommended that future research should develop and test the effectiveness of interventions that have potential to enhance carers’ participation in valued activities. The most recent study included in this review was published in 2015, and this field of research has grown considerably since that time. Further, as per the methodology, results were not adjusted based on methodological quality. As such, there is need for a systematic review of qualitative studies to understand the experiences of informal carers and the impact of role and activity changes on their health and wellbeing. By systematically synthesising qualitative research it can identify areas for future research and provide recommendations for evidence-based practice.

The purpose of this systematic review was to examine the experiences of informal carers and the impact of role and activity changes on their health and wellbeing. This review answers the following research questions:

1. What are the experiences of informal carers on the impact of providing care to stroke survivors?
2. How does caregiving impact on the roles and activities of stroke carers and subsequently their health and wellbeing?

## Methods

A qualitative interpretive approach was used within this review to focus on understanding the subjective meanings and experiences of informal carers. This review was reported in accordance with the Enhancing Transparency in Reporting the Synthesis of Qualitative Research (ENTREQ) guidelines [23] and Preferred Reporting Items for Systematic Reviews and Meta-Analyses (PRISMA) guidelines (see S1 Appendix). The review was prospectively registered in 2022 with the International Prospective Register of Systematic Reviews (registration number CRD42022335435). An initial search of CINAHL (EBSCO), MEDLINE (Ovid), Embase (Ovid), APA PsycInfo (EBSCO), and Web of Science was conducted in June 2022. A search strategy was developed using subject headings and keywords tailored to suit each database (see S2 Appendix). Searches were based on keywords related to population, study type, and outcomes. No organisations, websites, or clinical registers were searched. An updated search was conducted in October 2023.

### Study selection and data collection

Studies were eligible for inclusion in this review if they: (1) included informal stroke carers aged 18 years and over, regardless of the length of the caregiving role; (2) explored roles and valued activities of informal carers and/or consequences of activity change; and (3) used a qualitative methodology (i.e., qualitative data collection and analysis). Only studies published in English were included. No date limitations on studies were imposed to ensure all available studies were included. Studies were excluded if they included paid carers, or if stroke survivors lived in a residential care facility.

Within this review, valued activities were defined as any activity that is chosen by the informal carer and is of importance to them. This can include any kind of activity such as reading a book, socialising with others, or engaging in physical activity. Although providing care to others can be valued by informal carers, within the context of this review we did not consider these caretaking activities to be included as a ‘valued activity’.

Following database searches, records were identified and imported into Covidence [24]. Two authors (MJ and ND) independently screened the title and abstract of articles based on the selection criteria, and this was followed by full-text screening. This process was monitored by a third researcher (ESG) to resolve any conflicts. Reference lists of included studies were reviewed to screen for additional studies. Studies with more than one peer-reviewed publication based on the same sample were treated as one study.

### Data extraction and synthesis

Two reviewers independently extracted the data from each study including the study details (author, year of publication, and country), aim of the research, participant characteristics and sample size, phenomena of interest, and study results. To ensure key concepts and summaries were identified, the results from each study were considered as the text labelled as ‘results’, ‘findings’, or similarly titled sections of the published articles.

An interpretivist approach to the data synthesis was used to investigate and interpret the meaning of the data. Thematic synthesis was used to inductively analyse the extracted data from each study and identify recurring themes and patterns [25]. All results from studies were imported into NVivo 14 software for qualitative data analysis. The data synthesis comprised three stages: (1) line-by-line coding of the extracted data; (2) the ‘free codes’ were organised to construct categories of descriptive themes; and (3) the development of ‘analytical’ themes that went beyond the content of the included studies. One researcher (MJ) independently coded the results of all included studies, and a second researcher (ESG) independently coded the findings of 25% of the included articles chosen at random. During stage one and two of the analysis process the codes were organised into descriptive themes. Within these stages the researchers set aside the research questions to ensure they did not impose preconceived notions during analysis. Following this, within stage three one researcher (MJ) developed analytical themes and discussed these with another researcher (ESG) until consensus was reached. During this stage, to go beyond the findings of original studies the researchers used the research questions as a basis to guide the development of analytical themes. Based on carer perspectives presented in the included studies, researchers inferred enablers and barriers related to activity change and subsequent health and wellbeing outcomes. Any disputes were resolved by discussion with a third researcher (GSK).

### Critical appraisal

The Critical Appraisal Skills Programme (CASP) checklist was used to assess the methodological quality of included studies. The CASP checklist comprises 10 questions related to the rigour, credibility, and relevance of the study. Two researchers (MJ and ESG) conducted pilot testing on three studies to test the application of the CASP checklist for qualitative studies. Once pilot evaluation was completed, researchers (MJ and ESG) compared and discussed findings until a consensus was reached regarding the application and interpretation of the CASP. Each remaining study was then independently assessed and scored by two researchers (MJ and ESG) using the CASP checklist. Studies were not excluded based on CASP quality assessment, rather this checklist was used as descriptive information to add to the critical analysis and allow discussion around the credibility of findings.

## Results

**Fig 1:**
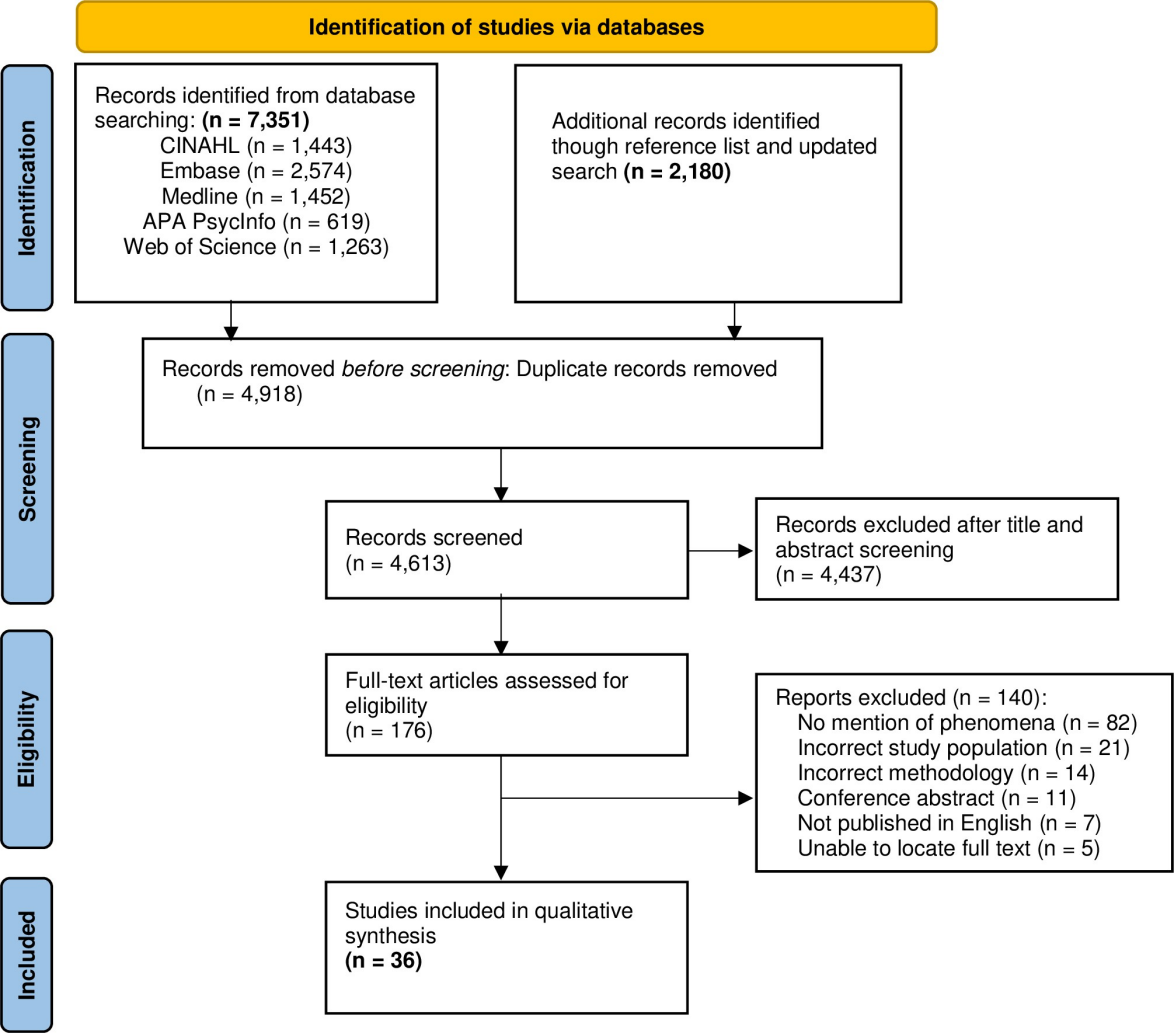
PRISMA flow diagram.

A total of 7,351 records were identified from database searches and an additional study [26] was identified and retrieved after reviewing the reference lists of included studies. After removing duplicates, two reviewers (MJ and ND) independently screened the title and abstract of the 7,351 records for eligibility. Of these records, the full text of 156 studies were retrieved and reviewed independently by two reviewers (MJ and ND). The most frequent reason for exclusion was not explicitly addressing carer activities, roles, or wellbeing. Three additional studies were identified through an updated database search in October 2023 [27–29]. Therefore, a total of 36 studies reporting on 34 unique samples met the inclusion criteria and were included in this review [7, 10, 12, 13, 26–57]. Fig 1 presents the screening process.

### Study characteristics

Characteristics of the included studies are presented in Table 1. The studies were published between 1997 [40] and 2023 [29] and three studies involved the participation of both stroke survivors and carers [30, 38, 48]. Studies were conducted across 12 countries, including the United States (n = 9), Canada (n = 5), United Kingdom (n = 6), and Australia (n = 4). A total of 601 carers were recruited across studies and the number of carer participants per study ranged from 3 [55] to 73 [27]. Of the studies that reported age, the mean age of carers ranged from 43.8 [29] to 65.5 years [37]. The majority of study participants were female (mean 80.9%), and 13 studies focused exclusively on female carers [13, 26, 29, 31, 33, 35, 39, 41, 43, 45, 50, 53, 55]. Data were collected via interviews (n = 28) [7, 12, 13, 26, 28–38, 40–44, 46–49, 51–55, 57], publicly available social media posts (n = 2) [27, 56], focus groups (n = 1) [39], questionnaires (n = 1) [50], or a combination of methods such as focus groups and interviews (n = 2) [10, 45]. The majority of studies collected data in person (n = 20) [10, 12, 28, 30, 31, 34, 36, 37, 39, 42–49, 52–55], with other data collection methods including online posts or platforms (n = 3) [27, 29, 56], via telephone (n = 2) [41, 58], or a combination of telephone and face-to-face (n = 6) [7, 13, 26, 33, 35, 40, 57]. The majority (n = 21) of the interviews and focus group ranged from 30 to 120 minutes in duration [10, 12, 13, 26, 29–33, 35, 37–39, 42–46, 52, 53, 55, 57].

**Table 1:** Table characteristics.

Study details	Study aim(s) (verbatim)	Sample	Participant characteristics	Data collection	Data analysis
Arntzen 2016 [30] Norway	This study explores stroke survivors’ and relatives’ negotiation of relational and activity change in their interrelated long-term meaning-making processes of everyday life and what it means for the experience of progress and well-being.	12	8 females. Age not reported. 5 partners, 2 siblings, 2 parents, 2 children, and 1 other relationship.	Semi-structured in-depth interviews, approximately 30–60 minutes in length. Interviews were conducted at a hospital, home, or at the researcher’s workplace.	Hermeneutic phenomenology
Aviles 2023 [29] Philippines	This study aims to address the following research objective, to explore the lived experiences of family caregivers of stroke survivors in Guadalupe, Cebu City	5	All females. Mean age 43.8 years (range 18–66). 2 partners, 1 child, 1 sibling, 1 grandchild.	Semi-structured interviews via an online video platform using an interview guide. Interviews were approximately 30–40 minutes.	Interpretative phenomenological design
Bäckström 2010 [31] Sweden	To illuminate the meanings of the middle-aged female spouses’ lived experience of the relationship with a partner who has suffered a stroke during the first year after their partner’s discharge from a rehabilitation clinic.	4	All females. Age range 40–58 years. All partners.	Narrative interviews were conducted at 1,6, and 12 months after the stroke patients discharge. The interviews lasted between 40–80 minutes and were conducted at the carer’s home or workplace.	Hermeneutic phenomenology
Barbic 2014 [32] Canada	This study describes a content validation of a conceptual model of emotional vitality in informal caregivers.	30	23 females. Mean age 54 years. 19 partners, 9 children, and 2 other relatives.	Semi-structured telephone interview, approximately 30–45 minutes in length.	Secondary thematic qualitative analysis using an inductive approach
Bastawrous 2014 [13]	To answer the following questions: What aspects of the pre and post stroke parent–child relationship contribute to daughters’ well-being? What changes, if any, do daughters perceive in the parent–child relationship over the course of their caregiving experience? How, if at all, do these changes contribute to daughters’ well-being?	23	All females. Median age 47 years (range 38–54). All daughters.	In-depth semi-structured interviews via telephone (n = 20) or in-person (n = 3). The interviews lasted a median of 46 minutes (range 40–62.5).	Thematic analysis
Bastawrous 2015 [33] Canada	To qualitatively explore daughters’ experiences with and response to holding multiple roles while providing post-stroke care.			In-depth semi-structured interviews via telephone (n = 21) or in-person (n = 2).	Thematic analysis
Bulley 2010 [34] United Kingdom	Carers’ experiences of caring for a stroke survivor were explored, including reactions and changes in their lives.	9	7 females. Age range 44–74 years. All partners.	Semi-structured interviews conducted at the carer’s home, approximately 23–68 minutes in length.	Interpretative phenomenological analysis
Buschenfeld 2009 [12] United Kingdom	To explore the experiences of partners of stroke survivors, the enduring effects, and changes of caring, and explore how caring influences psychological wellbeing.	7	3 females. Age range 49–62 years. All partners.	Semi-structured interviews conducted in the participants home (n = 6) or interviewers’ workplace (n = 1). Interviews lasted approximately 90 minutes.	Interpretative phenomenological analysis
Cao 2010 [35] Canada	Explore the perspectives of caregivers of persons with stroke with respect to their own physical activity.	10	All females. Age range 45–73 years. All partners.	Semi-structured in-depth interviews were conducted via telephone (n = 4) or face to face (n = 6). Interviews lasted approximately 1 hour.	Inductive thematic analysis
Cecil 2013 [36] United Kingdom	To explore caring and coping among carers of stroke survivors and identify factors that had an impact on their lives.	30	23 females. Age range 36–84 years. 19 partners, 7 children, 3 siblings, and 1 other relationship.	Semi structured interviews were conducted in the carer’s homes.	Thematic analysis
Coombs 2007 [37] Canada	The purpose of this study was to examine the experiences of spousal caregiving for stroke survivors.	8	5 females. Mean age 65.6 years (range 57–81). All partners.	Each participant engaged in 2 face to face semi-structured interviews which lasted approximately 60–120 minutes each.	Hermeneutic phenomenological analysis
de Leon Arabit 2008 [26] United States	The purpose of this study was to examine and explore the caregiving sentiments and coping strategies of five Latino women spouses of stroke survivors.	5	All females. Age range 57–85 years. All partners.	Interviews conducted via telephone (n = 3) or face-to-face (n = 2). Interviews lasted approximately 60 minutes.	Grounded theory—Constant comparative method
El Masry 2013 [38] Australia	The aim of this study was to explore the psychosocial aspects of the experiences, concerns, and needs of caregivers of persons following stroke.	20	16 females. Age range 31–90 years. 15 partners, 3 siblings, and 2 children.	Semi-structured interviews using an interview guide. Interviews were approximately 60 minutes.	Interpretive phenomenological analysis
Gosman-Hedström 2012 [39] Sweden	To explore and learn from the older women how they experience their life situation and formal support as carers of their partners after stroke and to suggest clinical implications.	16	All females. Median age 74 years (range 67–83). All partners.	Four focus groups with 3–5 participants each. Each group went for around 2 hours.	Thematic analysis
Grant 1997 [40] United States	To describe and categorize personal losses revolving around the self that caregivers experienced in caring for stroke survivors.	10	9 females. Age range 32–68 years. Relationship not reported.	Two semi-structured interview using open ended questions. First interview was conducted in the carer’s home or outpatient clinic. Second interview was conducted via telephone.	Grounded theory—Constant comparative method
Green 2009 [41] Canada	To explore perceptions of factors that impacted patients’ quality of life and wife caregivers’ strain over 12 months following minor stroke.	26	All females. Mean age 58.5 years (range 33–75). All partners.	Semi-structured interviews were conducted via telephone at 1,2,3,6,9, and 12 months post hospital discharge. Interviews ranged from 45–60 minutes.	Conventional content analysis
Greenwood 2010 [42] United Kingdom	Explores the impact on informal carers of caring for stroke survivors specifically focussing on the changes in carer autonomy and control and on the strategies they developed over the first 3 month to overcome the changes.	31	22 females. Age range 40- >65 years. 16 partners, 13 children, and 2 siblings.	Eighty-one in-depth interviews were conducted in the hospital or carer’s home. Carers were interviewed once (n = 4), twice (n = 4), or three (n = 23) times. Interviews ranged from 30–90 minutes.	Thematic analysis
Hodson 2020 [43] Australia	To answer the question: What is the essence of the mild stroke experience for the spouse during the first nine months after acute hospital discharge, in Australia?	4	All females. Aged ≥18 years. All partners.	Semi-structured interviews were conducted in participants homes and ranged between 40–74 minutes. An interview guide was used to guide conversation.	Interpretive phenomenological analysis
Johnson 1998 [44] United States	To investigate the lived experiences of rural caregivers of stroke survivors who have been in the caregiver role for at least 6 months poststroke.	10	5 females. Mean age 61.7 years (range 38–82). 6 partners, 3 children, and 1 daughter in-law.	Semi-structured interviews were conducted in the carer’s home using 7 open-ended probe questions.	Thematic analysis
Knecht-Sabres 2016 [45] United States	To explore the occupational needs of caregivers of stroke survivors and to identify potential implications for occupational therapy’s role with this population	4	All females. Mean age 65 years (range 50–75). All partners.	Semi-structured focus groups and individual interviews were conducted in a preferred location. Sessions lasted between 30–90 minutes.	Thematic analysis
Kniepmann 2014 [10] United States	To understand the occupational changes in spousal caregivers for survivors of stroke with aphasia.	12	10 females. Mean age 61.5 years (range 37–73). All partners.	Semi-structured interviews were conducted at a school campus, carer’s home, or community location. Sessions lasted approximately 40–90 minutes.	Inductive content analysis
Lobo 2023 [27] Australia	To identify needs specific to the caregiver and refine and evaluate these needs using an online-based survey.	72	58 females. Age range 24–83 years. 50 partners, 16 children, and 6 other relationships.	User posts on social media platforms such as Facebook and Twitter and an anonymous online survey.	Grounded theory
López-Espuela 2018 [46] Spain	To explore and document the experiences and values of spouse caregivers of stroke survivors. To gain more in-depth knowledge of how the act of caring and the adaption process affects caregiving spouses.	18	13 females. Mean age 55 years (range 42–80). All partners.	In-depth individual interviews and semi-structured interviews using a dynamic interview script were conducted with participants at a neurology office. Interviews lasted from 80–110 minutes.	Thematic content analysis
Lu 2019 [47]	The aim of this study was to explore the experience of family caregivers taking care of stroke survivors in China.	26	20 females. Mean age 63 years (range 27–82). 21 partners, 4 children and, 1 other relationship.	Semi-structured interviews using an interview guide were conducted at the carer’s home or at the hospital. Interviews lasted between 28–136 minutes.	Thematic analysis
Lu 2022 [28] China	This study aimed to deductively explore the needs of family members caring for stroke survivors in China.			Analysis of 26 interviews based on the Chinese version of the modified Caregiver Task Inventory.	Deductive content analysis
McCarthy 2015 [48] United States	The purpose of this study was to investigate the effects of stroke on couples, with a particular focus on how stroke may be experienced differently according to age and relationship duration.	31	18 females. Mean age 60.4 years (range 31–93). All partners.	Semi-structured interviews were conducted in person and lasted between 2–25 minutes.	Interpretive description
O’Connell 2004 [7] Australia	To determine carers’ perspectives of their support and educational needs while caring for the stroke survivor within the acute hospital, rehabilitation, and community settings.	37	23 females. Mean age 55.7 years (range 23–86). 21 partners, 13 children, and 3 other relationships.	Semi-structured interviews conducted face-to-face or via telephone and lasted approximately 20–30 minutes.	Thematic analysis
Rahman 2018 [49] Malaysia	To explore the experiences of caregivers in caring for the stroke survivors.	18	17 females. Mean age 50.6 years (range 25–73). 10 partners, 4 children, 3 sisters, and 1 other relationship.	Semi-structured interviews were conducted at the carer’s home (n = 12) or in a rehabilitative centre (n = 6).	Thematic analysis
Saban 2012 [50] United States	To describe the experience of female caregivers who care for an adult family member who has experienced a stroke within the previous year.	46	All females. Mean age 56.2 years (range 18–73). 24 partners, 18 children and, 2 other relationships.	Participants completed a written questionnaire containing open ended questions.	Grounded theory—Constant comparative method
Silva-Smith 2007 [51] United States	To develop a grounded theory that explained the process of preparing for and beginning a new caregiving role from the participants’ perspective.	12	9 females. Mean age 58 years (range 38–78). 7 partners, 2 siblings, 1 child, and 2 other relationships.	Twenty-three interviews were conducted with 12 participants.	Grounded theory—Constant comparative method
Simeone 2016 [52] Italy	To describe the lived experience of stroke caregivers three months following the discharge of patients from a rehabilitation hospital.	25	20 females. Mean age 55.9 years. 12 children, 10 partners, and 3 other relationships.	Interviews were conducted at the carer’s home using open-ended questions. Interviews lasted between 30–60 minutes.	Hermeneutic phenomenological analysis
Steber 2017 [53] United States	To further explore the perspective of caregivers of stroke survivors in terms of the value attributed to their leisure participation and its corresponding impact on their quality of life.	4	All females. Age range 55–80 years. All partners.	Semi-structured interviews conducted at the participants home (n = 2) or private location (n = 2). Interviews lasted between 60–80 minutes.	Transcendental phenomenology
Thomas 2008 [54] South Africa	Investigation into the complexities of caregiving, including both perceptions and experiences of the healthcare system.	6	4 females. Age range 16–67 years. 4 partners and 2 children.	A semi-structured interview using open ended questions was conducted at a clinic.	Content and thematic analysis
Van Dongen 2014 [55] Austria	Explored how some working Austrians experienced and coped with changes in their daily occupations after becoming informal carers of persons who had had a stroke.	3	All females. Age range 49–59 years. 2 partners and 1 child.	Two semi-structured open-ended interviews conducted at the carer’s home or therapy centre. Interviews lasted between 45–90 minutes.	Interpretive phenomenological analysis
Winkler 2014 [56] United Kingdom	This study explored how carers of people with aphasia perceive their roles and responsibilities.	10	9 females. Age not reported. 6 partners, 3 children, and 1 parent.	Nine publicly available carer blogs.	Passive analysis using “framework” method
Woodford 2018 [57] United Kingdom	To understand the specific difficulties experienced by caregivers experiencing elevated symptoms of anxiety and depression.	19	17 females. Mean age 57 years (range 21–77). 15 partners, 1 child, and 3 other relationships.	Semi-structured interviews using open-ended questions were conducted face-to-face or via telephone. Interviews lasted between 52–103 minutes.	Thematic analysis

### Synthesis

Four overarching themes were generated: (1) Life adjustment; (2) Changing role and identity; (3) Changing activities: From meaningful to purposeful; and (4) Understanding and supporting carers. Table 2 presents the overarching themes, subthemes, supporting extracts, and contributing studies for each theme.

**Table 2:** Themes and subthemes with selected quotes.

Themes and subthemes	Supporting quotes	Contributing studies
Life adjustment		
Acceptance and adaptation	“After all the lovely cards and the hot meals are delivered, there has to be personal, private ways that an individual deals with, grieves, and accepts what has happened.” [50, p.8]	[7, 10, 12, 27, 29–31, 35, 37, 38, 41, 42, 44–46, 48, 50–56]
Altered perceptions	“The loneliness is infinite. You feel so bad… I never wish for his death… but I do wish for mine… I’d like to disappear and let that be the end of it… we’ve made it thus far, and I can’t go on.” [46, p.11]	[7, 27, 28, 31, 32, 34, 35, 37, 41, 42, 46–48, 50, 51, 57]
Changing role and identity		
Relational changes	“We are more relaxed together. We spend more time together, eat dinner together and spend a long time at the dinner table talking. It’s very pleasant. Before he sat in front of the TV and I sat in the kitchen.” [30, p.44] “He’s not my husband anymore–we don’t have a relationship–I am his caregiver–our marriage ended with his stroke–he has his room and I have mine–his stroke and caregiving have affected my relationship with his children–they have alienated me.” [32, p.2869]	[7, 12, 13, 26, 27, 30–41, 43, 44, 46, 48, 50–52, 54–57].
Duty, obligation, and commitment	“I do not want to place him in a convalescent home. I do not feel he will be taken cared of well over there. I will continue to try to do my best to care for him at home as much as I can. This is where he will stay.” [26, p. 45] “I’m married, it is my duty.” [44, p.58]	[10, 13, 26, 27, 29–31, 36, 37, 44, 46–48, 54]
Sole responsibility	“We basically feel I’m working for two people, you know, I do Karen’s work, washing–although Karen does the ironing and does the dishes. I do the garden… and the house, and keep an eye on Karen.” [34, p.1409] ‘‘You feel that you have to take responsibility, you must carry it on your back.” [30, p.42] “I was terrified really, because I was worried I didn’t want him falling over, hurting himself, so I was maybe too mothering I suppose but I just wanted to make sure that he was safe”. [12, p.1645]	[12, 13, 26–32, 34–37, 39–57]
Losing sense of self	“My main and only role basically now, is a carer… I feel I haven’t got a life as such anymore. Not an individual life. My life revolves around my wife. I can’t do anything on the spur of the moment. I have no real individuality anymore.” [12, p.1647] “With his stroke—there is a point that you realize … maybe I’m not going to quite get back to the person I used to be.” [53, p.174] “So I don’t believe in leading a life of my own, I lead his life.” [57, p.298]	[12, 31, 40, 44, 46–48, 50, 53, 55–57]
Changing activities: From meaningful to purposeful		
Impact of activity change	"I get depressed sometimes because we used to travel a lot and go dancing. Now we are no longer able to do that. I get frustrated but I do not show it.” [26, p.45]	[7, 10, 12, 26–36, 38–57]
	“I studied at the Elderly University twice a week before, and I liked to draw every day when I was free. Now, I have given up the Elderly University. Where can I go? I cannot go anywhere, I cannot do anything I’m interested in. I spend all the time in with him. I do not have any time and energy to do anything else.” [47, p.5] “[I have] no uninterrupted time for personal things, including showers/baths, TV program or a movie, a chance to read or paint or play the organ. No chance to leave the house for a walk or to bike.” [50, p.6]	
Barriers to participation in valued activities	“You feel wrong doing it, because he can’t do it, and you know he’s thinking about you out there doing it and wanting to be there.” [35, p.40] “There just wasn’t enough hours in a day… it was very time consuming … I took care of him and I took care of the home. . . I was totally busy with my husband … there was no leisure time.” [45, p.4]	[10, 27–29, 36, 37–40, 42, 45, 47–50, 52, 53, 55–57]
Enablers to participation in valued activities	“So I have got a minder coming in twice a week and I go for three hours in the morning. It allows me to go to the bank as well as where I have got to get to, you know.” [42, p. 131] ‘‘I cast, he fishes and hooks the fish, I pull in, take off, bait and cast again. That’s how we do it.” [30, p.46] “The most important thing is that everyone around can laugh; and the other things. . . if we don’t do them today, we’ll do them tomorrow. I mean, you have to stand above things, you see, otherwise it wouldn’t be possible. . . . And it’s not that important how nicely this table is set.” [55, p.353]	[10, 26, 27, 30, 35, 41, 42, 45, 46, 48, 50–52, 55, 56]
Understanding and supporting carers		
Carer priorities	“I say yes, this has happened to you, but don’t you realise, are you not aware that involuntarily this has affected me? No, he does not want to see it. Even when I have had a low moment when I have cried, he tells me ‘I don’t understand why you’re crying. Why would you cry? ‘With what I’ve got going on, you’re going to cry on top of that?’ Do you understand? I can’t be neither fragile nor the martyr in all this.” [46, p.11] “She doesn’t realize she’s doing this, but she can be short and very demanding… you tend to feel like you’re being unappreciated or that you’re not doing a good enough job”. [13, p.1533] ‘‘We did not get as much help as we needed–I could have used more respite, especially at the beginning, my questions were poorly addressed by the [local community health clinic].” [32, p.2869]	[7, 10, 12, 13, 26–57]
Positive aspects of caring	“There’s an element of challenge and difficulty involved in it, but there is a high degree of privilege.” [44, p.59] ‘‘I value my partner more and other relationships more…The stroke put my life into perspective.” [41, p.1198]	[13, 26, 29, 38, 41, 43, 44, 52, 56]
Coping strategies	"God gives me the patience to take care of him (husband). I pray to Him (God) for my continued strength, patience and health so I can continue to take care of him.” [26, p.46] “I think the sense of humour keeps you going through it really, without that you haven’t got much hope in this life”. [12, p.1647]	[7, 10, 12, 26, 27, 30, 31, 35, 37–39, 43, 44, 46, 48, 50, 56]

#### Life adjustment

The theme of Life adjustment involved two subthemes: (1) Acceptance and adaptation and (2) Altered perceptions.

*Acceptance and adaptation*. The sudden and unexpected nature of stroke often required rapid readjustment in the life of the carer. This was evident across studies where participants discussed the need to accept their caregiving role and life changes associated with this [7, 12, 27, 44, 50, 53, 55, 56]. The acceptance of the caregiving role was often followed by adjustment and adaptation to the daily routines, activities, and arrangements. Across studies, carers frequently discussed the need to adjust to their new life situation [7, 10, 12, 27, 29–31, 35, 37, 38, 41, 42, 44–46, 48, 50–55]. To adjust to the new situation, some carers reported modifying their sleeping arrangements, completing activities in the morning, or staying up late at night to be able to complete activities [37, 42, 46, 49, 51]. Within one study, carers described adapting their activities such as using online shopping instead of attending the grocery store and meeting up with friends in a location closer to their home [42].

*Altered perceptions*. The caring role often resulted in carers needing to alter their future plans and perspectives [7, 27, 31, 32, 34, 35, 37, 41, 42, 46–48, 50, 51, 57]. Across studies, many participants discussed the uncertainties regarding the future for themselves as well as for the stroke survivor [7, 27, 37, 41, 42, 46–48, 50, 57]. For some carers, they reported feeling uncertain about their own health [46, 57], concerns regarding the possibility of the person they were caring for having a second stroke [27, 41, 50], and whether the stroke survivors’ function would improve [42, 47, 50, 57]. The life adjustment and uncertain future led some carers to consider death of the stroke survivor or themselves as a form of ‘liberation’ [28, 31, 46, 47]. These thoughts were associated with guilt and were deemed by carers as taboo to discuss [31, 46].

#### Changing role and identity

The theme of Changing role and identify included four subthemes: (1) Relational changes; (2) Duty, commitment, and obligation; (3) Sole responsibility; and (4) Losing sense of self.

*Relational changes*. Across most studies, carers discussed the impact of their caregiving role on their relationships with others such as the stroke survivor, family members, and friends [7, 12, 13, 26–28, 30–41, 43, 44, 46–48, 50–52, 54–57]. Participants suggested that becoming a carer resulted in a shift and change in the predefined roles or expectations in the relationship [12, 13, 27, 31, 32, 37–39, 46, 48, 50, 55–57]. This was evident for many participants providing care for their partner or parent who reported a clear shift in their relationship with the stroke survivor, evolving into a relationship that resembled that of a stranger, friend, or parent [12, 13, 31, 32, 37–39, 46, 55–57]. As a result, carers often reported experiencing a sense of relationship loss [12, 13, 31, 32, 35, 37–40, 44, 46, 50, 56, 57]. In addition, across studies carers suggested the changes in their relationship with the stroke survivor also led to loss of emotional support, intimacy, an increase in arguments, and lack of communication [12, 13, 27, 30–32, 37, 38, 40, 44, 46, 48, 50, 56]. Within studies, many carers reported experiencing frustration due to the change in the stroke survivors’ personality, cognitive, and emotional functioning, which also significantly impacted on their relationship with the stroke survivor [13, 26, 31, 32, 35, 37, 39, 43, 46, 50, 54].

Despite many of the relationship-based negative impacts that were reported, in some studies, carers also reported positive changes in their relationships with the stroke survivor and their family [12, 13, 30, 36, 38, 41, 44, 46, 48, 50, 52, 54, 56]. These positive changes included increased sense of unity and closeness with the stroke survivor [12, 13, 35, 38, 44, 46, 48, 52, 55, 56], improved communication [30, 41, 46], a stronger family bond [27, 36, 38, 41, 52, 54], spending more time with the stroke survivor [13, 30, 46, 48, 55, 56], and a greater appreciation of their relationship [35, 41, 50, 52, 55].

*Duty*, *commitment*, *and obligation*. Across 14 studies, carers reported that their reasons for taking on the caregiving role were related to a sense of obligation or duty and commitment to the stroke survivor [10, 13, 26, 27, 29–31, 36, 37, 44, 46–48, 54]. Within 11 of these studies, participants who were providing care for their parents or partner discussed a sense of responsibility and obligation to provide care as a spouse or child, or to “give back” to the stroke survivor [10, 13, 26, 27, 29–31, 36, 37, 44, 46]. Another reason for taking on the caregiving role was carers often reported feeling a strong sense of moral obligation or the need to fulfil their commitment to the stroke survivor [10, 13, 26, 31, 37, 44, 46]. In some studies, carers felt they were the best suited for the caregiving role or could provide better care than others [26, 27, 47], while in other studies, carers reported feeling as though they had no other choice or no one else could take on the caring role [29, 36, 37, 54]. Within four studies, participants discussed becoming a carer due to expectations from family members or society [13, 29, 30, 47]. Additionally, carers within four studies reported taking on the caring role to ensure the stroke survivor could continue to live at home rather than being placed in a long-term care facility [13, 26, 37, 44].

*Sole responsibility*. A common finding across studies was the assumption that the caregiving role often resulted in additional responsibilities [12, 13, 26–37, 39–57]. Across 17 studies, carers reported having to do “everything” on their own [26, 27, 30–32, 34, 36, 41, 44, 47–52, 55, 57]. These responsibilities included home maintenance [30, 32, 34, 35, 37, 40, 45, 47, 56], financial management [32, 45, 55, 56], meal preparation [30, 40, 47, 55, 56], and providing emotional support [48, 56] in addition to caring responsibilities [12, 13, 26–37, 39–57]. Carers often reported that there was a shift from shared responsibility to now being solely responsible for many tasks [27, 30–32, 34–37, 43, 50–52, 55–57]. Many carers identified that due to their caregiving role they were solely responsible for the care and decisions of the stroke survivor [27, 28, 32, 34, 41, 43–45, 47, 48, 50, 52, 56, 57], resulting in a state that was described as one of hypervigilance where many carers discussed having to be available and alert to the needs of the stroke survivor [27, 31, 37, 39, 41, 42, 45, 48, 50–52, 56, 57]. Within studies, participants discussed the additional responsibility of not only completing their own tasks but also those of the stroke survivor [30, 31, 47], this was labelled as ‘double duty’ within one study [37].

Across studies many carers reported that the additional roles and responsibilities impacted on their own health and wellbeing such as an increase in stress and anxiety, feeling overwhelmed, exhaustion, and isolation [7, 27, 30, 32, 33, 41, 56, 57]. Within one study, the concept of “role overload” was identified, indicating that carers often manage multiple roles and responsibilities [33]. Within 17 studies, participants reported that the caregiving role became their main priority resulting in the subsequent loss of other roles [12, 27, 30–32, 34, 40, 44–46, 49, 50, 52, 53, 55–57]. Within one study this concept was discussed as the “caring role takes over” [30].

*Losing sense of self*. The caregiving role was often described by participants as a ‘full-time’ job which impacted on their ability to maintain their previous roles such as being a parent [29, 33, 50, 51, 57], grandparent [27, 50], partner [27, 33, 44], child [44], friend [27, 50, 51], or employee [7, 32, 33, 36, 49]. Across studies, carers reported having to “juggle” multiple roles [32, 33, 44, 51, 53] which often resulted in what they described as uncertainty in their identity [12, 31, 40]. Some carers reported their loss of identity and meaning due to assuming the caregiving role which became the predominant role in their life [12, 31, 40, 46, 47, 53, 56, 57]. As a result of the demands of the caregiving role, and inability to fulfill previous roles, carers reported a loss in their individuality and, in some studies, carers questioned the meaning of their life [12, 31, 40, 44, 46–48, 50, 53, 55–57].

#### Changing activities: From meaningful to purposeful

The theme of Changing activities: From meaningful to purposeful included three subthemes: (1) Impact of activity change; (2) Barriers to participation in valued activities; and (3) Enablers to participation in valued activities.

*Impact of activity change*. Across studies carers discussed the change in their activities since assuming the caregiving role, which resulted in an increase in participation in care-related activities as opposed to what they described as valued activities [30, 35, 40, 42, 45, 53, 55, 57]. In one study, carers discussed completing activities that were necessary as opposed to what they would like to complete [53]. Across five studies, carers reported their activities changed to be more of a rehabilitative nature as opposed to recreational [27, 30, 35, 45, 56].

Within the majority of the studies (n = 21), participants discussed that most of their activities now included completing care related activities such as assisting the stroke survivor with feeding, dressing, showering, toileting, transfers, and therapeutic activities (i.e., stretches, prescribed exercises) [7, 13, 26, 27, 30, 34, 35, 37, 39, 41–43, 45, 46, 48–50, 52–54, 56]. The impact of caring activities on carer health and wellbeing was noted in 24 studies [12, 13, 26–29, 32–41, 45–47, 49–51, 54, 56]. This included impacts on physical health such as higher levels of fatigue [13, 26, 29, 34, 36, 37, 39, 50, 54], reduced sleep [13, 28, 32, 34, 36, 37, 41, 45, 47, 49, 51, 56], increase in blood pressure [32, 36, 40], weight loss [13, 26, 32, 45, 56], pain [13, 28, 32, 36, 45, 56], care-related injuries [45], reduced food intake [13, 56], and exacerbation of chronic health symptoms [26, 29, 34, 38]. Across 21 studies carers also reported the impact of caring on their emotional health such as feelings of anxiousness [27, 28, 32, 40, 41, 47], stress [13, 27–29, 32, 37, 40, 41, 45, 47, 54, 56], feeling depressed [26, 32, 34, 38, 47], anger [27, 28, 47], frustration [26, 27, 36, 50], and panic attacks [34, 36, 54].

Across 34 studies, carers discussed having to cease or reduce participation in valued activities to focus on caring for the stroke survivor [7, 10, 12, 26–36, 38–57]. Activity loss was frequently reported across several areas including social activities (n = 20) [10, 12, 26, 30–32, 34, 36, 38, 39, 41, 42, 44, 49, 50, 52–56], leisure activities (n = 18) [25–27, 32–35, 42–45, 47–50, 53, 55, 57], and employment (n = 16) [7, 12, 26, 32–34, 36, 38, 46–49, 52, 54, 55, 57]. Across seven studies, carers discussed how the caregiving role impacted on their ability to complete their own health-related activities [32–35, 50, 52, 57].

Across 20 studies, participants discussed the loss or change to their participation in social activities [10, 12, 26, 30–32, 34, 36, 38, 39, 41, 42, 44, 49, 50, 52–56]. Carers in these studies perceived various repercussions of the caregiving role on their social life and ability to complete social activities, including loss of friendships [38, 39], difficulty scheduling time to socialise [34, 41, 42, 49, 50], and a lack of social contact from friends [12, 36, 41, 44, 50, 53]. The caregiving role and loss of activities impacted on their emotional wellbeing as carers described feelings of loneliness and social isolation [12, 27, 34, 38, 41, 47, 48, 57]. For the few carers who were able to maintain some of their social activities, this was reported to be a coping strategy and positively impacted on their wellbeing and quality of life [26, 50, 55].

Among 18 studies, carers reported a reduction or cessation of leisure activities such as traveling, dancing, studying, camping, arts and crafts, and attending movies [25–27, 32–35, 42–45, 47–50, 53, 55, 57]. Across 10 studies, the impact of reduced participation in leisure activities was reported to impact on carer health and wellbeing in ways including increased stress, sadness, frustration, and feeling confined [26, 32, 35, 39, 45, 47, 51, 53, 54, 57]. The lack of spontaneity, autonomy, and freedom to complete preferred activities was noted in nine studies [27, 35, 40, 44, 47, 50, 53, 55, 57]. Within 10 studies, carers discussed feeling trapped and confined to their house [7, 27, 32, 37, 40, 42, 44, 51, 53, 57], and within some studies this was described by carers as feeling like a “prisoner” in their own life or home [34, 46, 47, 57].

Carers reported ceasing or reducing their employment due to the physical and emotional demands of caring, this was noted among 15 studies [7, 12, 26, 32–34, 36, 38, 46–49, 52, 54, 55, 57]. As a result, carers experienced financial implications impacting on their ability to pay bills, purchase desired and necessary items, and affording the various costs associated with caregiving such as rehabilitation services [7, 27, 28, 32, 38, 47–49]. For carers who ceased employment, this was often done unwillingly and resulted in resentment, frustration, or anger [7, 33, 46, 57]. Those carers who maintained employment reported an increase in stress and impact on their wellbeing due to managing both caring activities and employment [7, 27, 32, 36, 49]. For other carers, employment was viewed as a crucial coping strategy to promote their mental and emotional wellbeing [12, 55].

A total of 10 studies reported on the impact of the caregiving role on their ability to complete health-related activities [28, 32–35, 45, 50–52, 57]. Carers discussed the reduction or cessation of activities such as playing sports [32, 33, 51], going for walks [34, 35, 50, 52, 57], exercising [28, 33, 35, 45, 51], and attending medical appointments [32].

*Barriers to participation in valued activities*. Across 20 studies, carers discussed the barriers to participation in valued activities [10, 27, 29, 30, 35, 37–40, 42, 43, 45, 47, 49, 50, 52, 53, 55–57]. Across studies the most commonly reported barrier to engaging in valued activities was viewing the care and needs of the stroke survivor as a priority [27, 29, 30, 37, 39, 40, 42, 45, 47, 49, 50, 53, 55, 57]. Within one study, this was described as carers “realising that the reduction of some occupations was necessary to manage the situation” [55]. Within other studies, carers reported that the effects of stroke such as reduced mobility, cognitive deficits, and emotional changes impacted on their ability to participate in activities with the stroke survivor or leave the stroke survivor at home to engage in activities [10, 27, 35, 42, 45, 57].

The lack of time and energy as a barrier to participating in meaningful activities was reported in 12 studies [10, 27, 35, 37, 39, 40, 43, 45, 47, 50, 52, 56]. Carers within three studies discussed the lack of “uninterrupted” time to pursue activities of interest [39, 50, 55]. Another barrier discussed across studies related to a lack of desire to participate in activities without the stroke survivor [10, 35, 42]. Within four studies carers reported feeling guilty when participating in valued activities when the stroke survivor could not also participate [29, 35, 37, 42]. Financial strain was reported within three studies as a barrier to participating in valued activities as carers often as a result had to reduce social and recreational activities [10, 38, 57].

*Enablers to participation in valued activities*. Enablers to participation in valued activities were reported in 14 studies [10, 27, 29, 30, 35, 41, 42, 45, 47, 48, 51, 52, 55, 56]. The carers’ ability to adapt and reprioritise their activities as an enabler toward participation in meaningful activities was noted in eight studies [10, 30, 35, 41, 45, 48, 51, 55]. Within studies, carers discussed strategies such as changing the time they complete an activity [45], prioritising shared activities with the stroke survivor [30, 35, 45, 48, 55], and rethinking priorities [32, 41]. Within one study carers discussed the importance of “being” with the stroke survivor which was prioritised over household tasks [55]. Within two studies carers discussed prioritising a particular activity such as employment over other activities [32] or refocusing on the meaning of family and relationships [41]. Carers within one study discussed the importance of re-evaluating priorities and used strategies such as travelling on motorised scooters and dancing in the living room with the stroke survivor [38]. Within three studies carers reported changing the way they completed their activities to enable participation, such as reducing the size of their garden, [55] using accessible campsites [30], or completing online shopping [42].

Across six studies, strategies such as planning, developing a routine, and being flexible were reported to enable participation in valued activities [10, 38, 39, 42, 45, 51]. Carers reported that planning ahead and taking precautionary measures facilitated success in resuming valued activities with the stroke survivor [39, 42]. Within two studies planning involved consideration of bus routes, availability of ramps, and sufficient circulation space for a wheelchair [42, 56].

Across five studies, it was noted that with time and recovery of the stroke survivor, carers were able to gain experience which allowed reengagement in valued activities [29, 35, 45, 51, 55]. Within two studies the recovery or improvement of the stroke survivor resulted in carers being able to resume valued activities such as travel [35, 52, 55]. The use of reliable paid and unpaid support was reported as a facilitator in seven studies which allowed carers to engage in valued activities [26, 27, 35, 42, 46, 51, 56].

#### Understanding and supporting carers

The theme of understanding and supporting carers included three subthemes: (1) Carer priorities; (2) Positive aspects of caring; and (3) Coping strategies.

*Carer priorities*. The most frequent priority discussed across studies (n = 30) was the need for support, training, and services to assist with the caregiving role [7, 10, 12, 13, 26, 27, 29, 30, 32–40, 42–47, 50–52, 54–57]. Across eight studies, carers identified that lack of information regarding the emotional and physical impact of assuming the caregiving role often resulted in carers feeling unprepared and ill equipped [27, 32, 34, 36, 39, 45, 50, 52]. Within two studies, carers reported that health professionals often provided limited information and communication in the form of brochures and pamphlets which were often not useful [27, 36]. In some studies, carers discussed the need for financial and resource support from the healthcare system such as respite services [27, 32, 38, 45, 50, 56]. For carers who had access to paid support services, they reported that these services were often unreliable, had a high turnover rate, or did not provide quality care [42, 50]. Carers identified the need for realistic and useful strategies to implement in their daily life and information provided in a user-friendly or worthwhile manner rather than “generic” information, this was noted in four studies [27, 36, 50, 57]. Carers reported within two studies that they would like health professionals to instil hope rather than have a “pessimistic” outlook [38, 50]. Across five studies carers identified the importance of carer support groups to engage with others with similar experiences who can understand, listen, and talk through caring experiences [13, 27, 45, 50, 57]. Within one study a participant reported “there’s a lot of stuff to make the caregiver a better caregiver, but not necessarily to make the caregiver’s life any better” [45].

Across studies (n = 11) carers discussed how the caregiving role often left them feeling alone, misunderstood, and underappreciated [13, 27, 28, 30, 32, 38, 46, 47, 50, 51, 56]. Carers reported that despite their significant efforts, they rarely received appreciation or expressions of gratitude, and this led to reduced self-worth and a sense that their contributions to the care of the stroke survivor were insufficient [13, 50, 56]. As a result, carers identified the need to be understood and recognised for their role and efforts [27, 28, 30, 46, 47, 51, 57].

Another priority that was identified across a large number of studies (n = 28) was the need for time and space to relax and switch off from the caregiving role [7, 12, 27–32, 35, 38–47, 49–51, 53–57]. Carers frequently reported that they did not have time to relax and complete their own personal activities. Switching off involved carers finding time or moments to independently pursue activities of interest such as participating in social outings with friends or watching television.

*Positive aspects of caring*. Across nine studies, carers reported some of the positive aspects of caring and their satisfaction with their caregiving role [13, 26, 29, 38, 41, 43, 44, 52, 56]. Within these studies carers expressed a sense of accomplishment and a positive outlook on the value and impact of their caregiving role. Within five studies, carers identified that their role allowed them to make a meaningful contribution to the life of the stroke survivor [13, 26, 29, 44], and as a result, for some carers their role was viewed as a privilege [36, 44]. Some additional positive aspects of caring that were discussed included an increased appreciation for life and mastering new skills and knowledge [38, 41, 44, 50, 56].

*Coping strategies*. Within 23 studies, carers discussed strategies they used to cope with their caregiving role. These strategies involved faith, spirituality, and religion (n = 7) [10, 26, 27, 37, 40, 46, 50], use of humour (n = 5) [7, 12, 30, 48, 56], and positive thinking (n = 11) [7, 26, 30, 31, 35, 37–39, 43, 44, 56]. Carers identified that their faith in God, use of prayer, and strong spiritual life provided them with patience, hope and resilience to cope with uncertainties and their caregiving role [10, 26, 27, 37, 40, 50]. In addition, humour was used as a strategy to assist carers to cope with challenging situations [7, 12, 30, 48, 56]. Maintaining optimism and hope was reported as another strategy to assist carers to cope with their caregiving role [7, 31, 37, 39, 44, 56]. Carers often tried to maintain a positive outlook of their future and the stroke survivors’ recovery.

In summary, it was evident that the sudden and expected nature of stroke often required life adjustment for carers which subsequently led to changes in their roles and identity. Due to the changes in roles and identity, carers often reported prioritising their caring role which led to their engagement in more purposeful activities as opposed to meaningful. Overall, the changes to life, roles, identity, and subsequently valued activities had a significant impact on the health and wellbeing of carers. However, it was evident that carers discussed strategies and their needs which contribute to their health and wellbeing.

### Methodological quality of included studies

The results of the CASP assessment can be found in S3 Appendix. All studies provided a clear statement of the research aim, the selection of qualitative methodology was appropriate, and the research design was justified and deemed suitable to address the research goal. Five studies did not provide information regarding participant selection and recruitment. All studies justified their methods and indicated how they collected data in a way that addressed the research issue. Within 17 studies, researchers did not report critically examining or considering the role between the researcher and participants. The majority of studies (n = 31) provided sufficient detail on how researchers adhered to ethical procedures and considered ethical issues such as obtaining ethics approval, written informed consent, and advising participants they can withdraw at any time. For all studies, there was adequate description of the data analysis process and a clear statement of findings.

## Discussion

This review aimed to gain an in-depth understanding of the impact of caring on the activities and roles of carers and how these changes impact on their health and wellbeing. Using a thematic synthesis approach four overarching themes were identified: (1) Life adjustment; (2) Changing role and identity; (3) Changing activities: From meaningful to purposeful; and (4) Understanding and supporting carers. This review identified changes in the roles and responsibilities of carers which subsequently impacted on their relationships, identity, and meaning of life. The caregiving role was discussed as a priority that can influence the type of activities carers participate in, such as completing more purposeful activities as opposed to recreational activities. As a result, carers reported changes in their physical and mental health as well as financial strain.

In line with the findings of this systematic review, previous reviews have identified the implications associated with providing care for a stroke survivor, such as managing multiple roles and responsibilities and changes to relationships [59–61]. A review by Greenwood et al. [60] found that the caregiving role can impact on carers’ relationships and result in uncertainty about the future. Similarly, a recent systematic review by Kokorelias et al. [59] found that carers restructure their life to accommodate the needs of the stroke survivor. The present review also found the demands of caregiving can impact on the physical health and psychosocial health of carers. Within the current review it is evident the caregiving role can impact on the type of activities carers participate in as well as the level of engagement in these activities. A scoping review by Jellema et al. [22] explored activity change for stroke caregivers and found that although there is a lack of evidence in this area, sustained activity loss can result in poorer health and wellbeing outcomes for carers. Jellema et al. [22] also identified a lack of intervention studies that assist carers to maintain their valued activities.

Uniquely, our review highlighted the enablers and barriers that can impact on carers participation in valued activities. Strategies such as planning, adapting activities, developing a routine, and prioritising activities were identified as enablers to participating in valued activities. Whereas factors such as lack of time and energy, prioritising the needs of the stroke survivor, financial strain, and feelings of guilt were identified as barriers to carer participation in valued activities. A study by Arksey [62] developed and tested a model of support for working carers and included strategies to support employment such as leave policies, carer-friendly work arrangements, access to a private telephone, and supportive supervisors and colleagues. Arksey [62] suggests that there is no single solution or strategy to supporting carers in their workplace. The current review further contributes to this research by identifying potential strategies in maintaining valued activities beyond employment.

The studies within this review recruited carers at different time points in their caregiving role or did not report the length of caregiving. It is evident that the stroke caregiving trajectory can be nonlinear and due to the lengthy and often uncertain recovery of the stroke survivor it can be difficult to predict the needs and outcomes of carers over time. Evidence suggests that carers often experience a decline in their valued activities shortly after the stroke onset [22]. A reduction in leisure and social activities is associated with increased stress, depression, and decreased life satisfaction [63–65]. Studies within other carer populations (i.e., dementia) have found that engaging in social or leisure activities can act as a protective factor against the detrimental effects of caregiving [66] and improve mental and physical wellbeing for carers [63, 67]. Therefore, it is important that where possible, interventions incorporate strategies to maintain valued activities and are designed to suit the specific needs of carers at different time points in the caregiving trajectory.

Within this review, the positive aspects of caring and carer needs and priorities such as additional support, time to relax, and recognition were identified. Previous studies have tested the effectiveness of interventions to address these carer needs and priorities. A study by Wang et al. [68] tested the effectiveness of a muscle relaxation program for carers, whereas Eames et al. [69] provided carers with an educational support program. However, neither of these interventions were effective in improving carer outcomes (caregiver strain, burden) post intervention. A study by Fu et al. [70] delivered a benefit finding intervention where carers were encouraged to discover the positive aspects of caring and found improvement in caregiver burden outcomes post intervention. It is evident, however that well designed and fully powered trials are still needed to confirm the efficacy of these interventions. Further interventions are still required to address unmet carer needs, such as enabling participation in valued activities that promote overall health and wellbeing.

### Strengths and limitations

A particular strength of this study was the focus on the impact of caregiving on the roles and valued activities of stroke carers. This review is the first to our knowledge to report on the enablers and barriers to participating in valued activities and strategies that may be effective to maintain participation in valued activities for stroke carers.

This review also had some limitations. First, although this review included a variety of perspectives from 12 countries, only articles published in English were included. As a result, the findings may not represent the diversity of carer experiences within different cultures. Similarly, grey literature was not searched which may exclude relevant studies not published in journals. Third, there is debate around whether the use of critical appraisal tools is useful for qualitative studies and what constitutes “validity” and “quality” within these studies [71, 72]. Despite this, the importance of using critical appraisal tools is still recommended [71, 72]. To address this limitation, critical appraisal was conducted independently by two reviewers and reporting standards were utilised to ensure transparency of research methods.

### Recommendations

It is recommended that health professionals and researchers collaborate with stroke carers to identify their valued activities and develop realistic strategies to maintain participation in these activities. Health professionals should support carers to use strategies discussed within this review to enable participation in valued activities. These strategies include planning ahead, adapting or seeking out alternative activities to suit their needs, developing a routine, and prioritising activities. Future interventions designed for carers should incorporate education for carers about the importance of maintaining valued activities and practical strategies they can implement to maintain these activities.

It remains unclear what type of valued activities are vital to maintain or prioritise. Therefore, future qualitative research is required to explore how to best support carers to maintain participation in their valued activities and when this support would be most beneficial. It is also recommended that future qualitative studies report details related to recruitment and data collection methods, as well as critically examining the role of the researcher and relationship between researcher and participants.

## Conclusion

The findings of this review add to the understanding of the complexity of caring emphasising the sudden and uncertain nature of stroke. This review further highlights the transition to becoming a carer requires major life adjustment that has implications on the carer’s relationships, roles, and activities, subsequently impacting on the health and wellbeing of the carer. Health professionals should provide carers with support to identify their valued activities and implement strategies to maintain and prioritise these valued activities. There is also a need for future carer interventions to incorporate education and strategies to promote participation in valued activities. This, in turn, may improve carer outcomes such as health and wellbeing.

## Supporting information

S1 Appendix PRISMA checklist. (DOCX)

S2 Appendix Search strategy. (DOCX)

S3 Appendix Quality appraisal of included studies. (DOCX)
