# Supplementary material for: The impact of caregiving on the roles and valued activities of stroke carers: A systematic review of qualitative studies
Source: PLoS One. 2024 May 31;19(5):e0304501. doi: 10.1371/journal.pone.0304501 (PMC11142509; doi:10.1371/journal.pone.0304501)
Supplement: S2 Appendix — (DOCX) [file pone.0304501.s002.docx]

| S1 | Carer* OR Care-giver* OR Caregiver* OR "informal care*" OR "family care*" OR "Primary care*" OR spouse* OR parent* OR Partner* OR Relative* |
| --- | --- |
| S2 | Participation OR meaningful OR Activit* OR Role* OR Leisure OR Social OR “Life experienc*” OR Occupation* OR “Activity change” OR Wellbeing OR Well-being OR Health OR "Quality of Life" OR Stress OR Strain OR Burden OR Burnout OR Depression OR Anxiety OR Overload OR Experience* OR Support OR Coping OR Psychological OR Performance OR Satisfaction OR attitude* OR perception* OR experience* OR lifestyle or engagement |
| S3 | Stroke OR CVA OR (cardiovascular W2 (accident* OR event*)) ) |
| S4 | Qualitative OR “Focus group*” OR Interview* OR “Grounded theory” OR Phenomenolog* OR “Content Analysis” OR “Thematic Analysis” OR Theme* OR Ethnograph* OR Mixed-Method* |
| S5 | S1 + S2 + S3 + S4 |

**S2 Appendix. Search Strategy**
