# Supplementary material for: The impact of caregiving on the roles and valued activities of stroke carers: A systematic review of qualitative studies
Source: PLoS One. 2024 May 31;19(5):e0304501. doi: 10.1371/journal.pone.0304501 (PMC11142509; doi:10.1371/journal.pone.0304501)
Supplement: S3 Appendix — (DOCX) [file pone.0304501.s003.docx]

**S3 Appendix. Quality appraisal of included studies.**

| **Study details** | **CASP Criterion 1**  Clear statement of aim | **CASP Criterion 2**  Qualitative methodology appropriate? | **CASP Criterion 3**  Appropriate research design | **CASP Criterion 4**  Appropriate recruitment strategy | **CASP Criterion 5**  Data collection | **CASP Criterion 6**  Relationship between the researcher and participants | **CASP Criterion 7**  Ethical issues | **CASP Criterion 8**  Data analysis and rigour | **CASP Criterion 9**  Clear statement of findings | **CASP Criterion 10**  Value of research |
| --- | --- | --- | --- | --- | --- | --- | --- | --- | --- | --- |
| Arntzen 2016 | Yes | Yes | Yes | Yes | Yes | Yes | Yes | Yes | Yes | Valuable |
| Aviles 2023 | Yes | Yes | Yes | Yes | Yes | Yes | Yes | Yes | Yes | Valuable |
| Bäckström 2010 | Yes | Yes | Yes | Yes | Yes | No | Yes | Yes | Yes | Valuable |
| Barbic 2014 | Yes | Yes | Yes | Unclear | Yes | No | Yes | Yes | Yes | Valuable |
| Bastawrous 2014 | Yes | Yes | Yes | Yes | Yes | Yes | Yes | Yes | Yes | Valuable |
| Bastawrous 2015 | Yes | Yes | Yes | Yes | Yes | Yes | Yes | Yes | Yes | Valuable |
| Bulley 2010 | Yes | Yes | Yes | Yes | Yes | Unclear | Yes | Yes | Yes | Valuable |
| Buschenfeld 2009 | Yes | Yes | Yes | No | Yes | No | Yes | Yes | Yes | Valuable |
| Cao 2010 | Yes | Yes | Yes | Yes | Yes | No | Yes | Yes | Yes | Valuable |
| Cecil 2013 | Yes | Yes | Yes | Yes | Yes | No | Yes | Yes | Yes | Valuable |
| Coombs 2007 | Yes | Yes | Yes | Yes | Yes | No | No | Yes | Yes | Valuable |
| de Leon Arabit 2008 | Yes | Yes | Yes | Yes | Yes | No | Yes | Yes | Yes | Valuable |
| El Masry 2013 | Yes | Yes | Yes | Yes | Yes | No | No | Yes | Yes | Valuable |
| Gosman-Hedström 2012 | Yes | Yes | Yes | Yes | Yes | Yes | Yes | Yes | Yes | Valuable |
| Grant 1997 | Yes | Yes | Yes | Yes | Yes | Yes | No | Yes | Yes | Valuable |
| Green 2009 | Yes | Yes | Yes | Yes | Yes | No | Yes | Yes | Yes | Valuable |
| **Study details** | **CASP Criterion 1**  Clear statement of aim | **CASP Criterion 2**  Qualitative methodology appropriate? | **CASP Criterion 3**  Appropriate research design | **CASP Criterion 4**  Appropriate recruitment strategy | **CASP Criterion 5**  Data collection | **CASP Criterion 6**  Relationship between the researcher and participants | **CASP Criterion 7**  Ethical issues | **CASP Criterion 8**  Data analysis and rigour | **CASP Criterion 9**  Clear statement of findings | **CASP Criterion 10**  Value of research |
| Greenwood 2010 | Yes | Yes | Yes | Yes | Yes | Yes | Yes | Yes | Yes | Valuable |
| Hodson 2020 | Yes | Yes | Yes | Yes | Yes | Yes | Yes | Yes | Yes | Valuable |
| Johnson 1998 | Yes | Yes | Yes | No | Yes | Yes | No | Yes | Yes | Valuable |
| Knecht-Sabres 2016 | Yes | Yes | Yes | Yes | Yes | Yes | Yes | Yes | Yes | Valuable |
| Kniepmann 2014 | Yes | Yes | Yes | Yes | Yes | No | Yes | Yes | Yes | Valuable |
| Lobo 2023 | Yes | Yes | Yes | Yes | Yes | No | Yes | Yes | Unclear | Valuable |
| López-Espuela 2018 | Yes | Yes | Yes | Yes | Yes | Yes | Yes | Yes | Yes | Valuable |
| Lu 2019 | Yes | Yes | Yes | Yes | Yes | Yes | Yes | Yes | Yes | Valuable |
| Lu 2022 | Yes | Yes | Yes | Yes | Yes | Yes | Yes | Yes | Yes | Valuable |
| McCarthy 2015 | Yes | Yes | Yes | Yes | Yes | No | Yes | Yes | Yes | Valuable |
| O’Connell 2004 | Yes | Yes | Yes | Yes | Yes | No | Yes | Yes | Yes | Valuable |
| Rahman 2018 | Yes | Yes | Yes | Unclear | Yes | No | Yes | Yes | Yes | Valuable |
| Saban 2012 | Yes | Yes | Yes | Yes | Yes | No | Yes | Yes | Yes | Valuable |
| Silva-Smith 2007 | Yes | Yes | Yes | Yes | Yes | No | Yes | Yes | Yes | Valuable |
| Simeone 2016 | Yes | Yes | Yes | Yes | Yes | Yes | Yes | Yes | Yes | Valuable |
| Steber 2017 | Yes | Yes | Yes | Yes | Yes | Yes | Yes | Yes | Yes | Valuable |
| **Study details** | **CASP Criterion 1**  Clear statement of aim | **CASP Criterion 2**  Qualitative methodology appropriate? | **CASP Criterion 3**  Appropriate research design | **CASP Criterion 4**  Appropriate recruitment strategy | **CASP Criterion 5**  Data collection | **CASP Criterion 6**  Relationship between the researcher and participants | **CASP Criterion 7**  Ethical issues | **CASP Criterion 8**  Data analysis and rigour | **CASP Criterion 9**  Clear statement of findings | **CASP Criterion 10**  Value of research |
| Thomas 2008 | Yes | Yes | Yes | Yes | Yes | Yes | Yes | Yes | Yes | Valuable |
| Van Dongen 2014 | Yes | Yes | Yes | Yes | Yes | Yes | Yes | Yes | Yes | Valuable |
| Winkler 2014 | Yes | Yes | Yes | Unclear | Yes | No | Yes | Yes | Yes | Valuable |
| Woodford 2018 | Yes | Yes | Yes | Yes | Yes | Yes | Yes | Yes | Yes | Valuable |
